# Evaluation of the anticancer effects of kaempferol using *in vitro* assays and [^99m^Tc]Tc-PSMA SPECT/CT imaging in a DMBA-induced breast cancer model

**DOI:** 10.3389/fphar.2026.1961418

**Published:** 2026-10-08

**Authors:** Fatma J. Al-Saeedi

**Affiliations:** Department of Nuclear Medicine, College of Medicine, Kuwait University, Shadadiya, Kuwait

**Keywords:** [^99m^Tc]Tc-PSMA, breast cancer, flavonoids, kaempferol, nuclear medicine, radiopharmaceuticals, SPECT/CT, tumor imaging

## Abstract

**Background:**

Kaempferol is a naturally occurring flavonoid with reported anticancer properties; however, its effects on breast cancer cells and tumor-associated molecular imaging require further investigation. This study evaluated the effects of kaempferol using complementary *in vitro* assays and a 7,12-dimethylbenz [a]anthracene (DMBA)-induced breast cancer rat model assessed by [^99m^Tc]Tc-PSMA SPECT/CT imaging.

**Methods:**

MCF-7 breast cancer cells were treated with kaempferol, and cell viability was assessed using the MTT assay across a concentration range of 0–80 µM. Apoptosis, wound closure, and the expression of E-cadherin and VEGF were also evaluated. The half-maximal inhibitory concentration (IC_50_) was estimated using nonlinear four-parameter logistic regression of replicate-level concentration–response data. *In vivo*, 8-week-old rats were allocated into cancer control, kaempferol pre-treatment, and post-treatment groups (n = 10/group). Mammary tumors were induced using DMBA, and animals received kaempferol (100 mg/kg/day, oral gavage) according to the experimental protocol. Tumor-associated tracer uptake was evaluated using [^99m^Tc]Tc-PSMA SPECT/CT imaging and quantified using tumor-to-background ratios (TBRs).

**Results:**

Kaempferol reduced MCF-7 cell viability in a concentration-dependent manner, with nonlinear regression estimating an IC_50_ of approximately 40 µM. Kaempferol treatment was associated with increased apoptosis, reduced wound closure, increased E-cadherin expression, and decreased VEGF expression. *In vivo* imaging demonstrated significant differences in tumor tracer uptake among the experimental groups. The pre-treatment group showed significantly lower TBR values (64.65 ± 1.69) than the cancer control (71.18 ± 1.83) and post-treatment groups (71.16 ± 1.06), whereas no significant difference was observed between the cancer control and post-treatment groups. One-way ANOVA demonstrated a significant overall group effect (F (2,27) = 58.08, p < 0.001, η^2^ = 0.81).

**Conclusion:**

Kaempferol demonstrated concentration-dependent cytotoxic effects and modulation of cancer-associated cellular markers in MCF-7 cells. In the DMBA-induced breast cancer model, reduced tumor-associated [^99m^Tc]Tc-PSMA uptake was observed predominantly following kaempferol pre-treatment, suggesting a potential chemopreventive effect. The findings support the complementary use of *in vitro* pharmacological assays and molecular imaging to investigate treatment-associated changes in tumor biology.

## 1 Introduction

Breast cancer remains the most frequently diagnosed cancer in women worldwide, accounting for approximately 2.3 million new cases and over 660,000 deaths annually (Jatoi, 2024; Breast cancer, 2025; Breast cancer statistics, 2025).

Despite advances in targeted therapies, treatment resistance and metastasis continue to limit clinical outcomes. Natural compounds, particularly flavonoids such as kaempferol, have gained attention due to their ability to modulate multiple oncogenic pathways.

Natural flavonoids have attracted considerable attention because they target multiple signaling pathways simultaneously, possess relatively low toxicity profiles, and may enhance the efficacy of conventional therapies while reducing adverse effects (Shen et al., 2022; Abdollahi et al., 2026).

Kaempferol is a natural flavonoid found in fruits, vegetables, and herbs with recognized anticancer and anti-metastatic properties (Periferakis et al., 2022; Herrera et al., 2025). Its effects include inhibition of cell proliferation and suppression of metastatic behavior across several cancer types, including breast cancer. Mechanistically, kaempferol modulates matrix metalloproteinases (MMPs) (Cho et al., 2023), cytoskeletal regulators (Khedgikar et al., 2016; Bolaños et al., 2015), signaling pathways (e.g., MAPK, Rho/Rac) (Miao et al., 2026), and cellular adhesion and invasion processes (Alvizo-Rodríguez et al., 2025; Wu et al., 2023; Hung et al., 2017). Kaempferol has been shown to induce apoptosis (Kubina et al., 2023), suppress epithelial–mesenchymal transition (EMT) (Zhang et al., 2025; Lee et al., 2017), and inhibit PI3K/AKT and NF-κB signaling pathways (Yu et al., 2026; Zhang et al., 2019). These mechanisms are critical in tumor progression and metastasis (Kaur et al., 2024; Kubatka et al., 2024).

Molecular imaging using technetium-99 m labelled with prostate-specific membrane antigen (PSMA) ([^99m^Tc]Tc-PSMA) has emerged as a promising tool for tumor detection (Janota et al., 2026; Li Q et al., 2025; Jonmarker et al., 2026). Although PSMA is primarily associated with prostate cancer, it is also expressed in tumor neovasculature of non-prostatic malignancies, including breast cancer (Takáč et al., 2023) (Abdel Maguid et al., 2023) (Mahzouni and Shavakhi, 2019; Liu et al., 2025).

MCF-7 cells were selected because they represent estrogen receptor-positive breast cancer, the most common molecular subtype of human breast cancer. Although MCF-7 cells exhibit relatively low invasiveness, migration and EMT assays remain useful for evaluating anti-metastatic mechanisms.

Although the anticancer properties of kaempferol have been investigated in various breast cancer models, little is known regarding its influence on PSMA-targeted molecular imaging signals *in vivo*. Furthermore, studies integrating functional nuclear imaging with flavonoid-based interventions remain limited. Therefore, the present study uniquely combines *in vitro* mechanistic evaluation with [^99m^Tc]Tc-PSMA SPECT/CT imaging in a DMBA-induced breast cancer model to investigate both therapeutic and imaging-related responses to kaempferol treatment.

## 2 Materials and methods

### 2.1 Chemicals and reagents

Kaempferol, 7,12-dimethylbenz [a]anthracene (DMBA), dimethyl sulfoxide (DMSO) [3-(4,5-dimethylthiazol-2-yl)-2,5-diphenyltetrazolium bromide] (MTT) reagents were purchased from Sigma-Aldrich Corp (St. Louis, United States). Guava Nexin apoptosis kit was purchased from Cytek Biosciences (Fremont, United States). PSMA I&T kit was obtained from The Center of Molecular Research (Moskva, Russia).

### 2.2 Kaempferol preparation

Kaempferol (purity ≥98%) was dissolved in dimethyl sulfoxide (DMSO) to prepare a 100 mM stock solution. The stock solution was aliquoted and stored at −20 °C until use. Before each experiment, appropriate dilutions were freshly prepared in complete cell culture medium to obtain the required final concentrations according to the specific experimental assay. For the MTT dose–response experiment, kaempferol concentrations ranging from 0 to 80 µM (0, 5, 10, 15, 20, 25, 30, 35, 40, 45, 50, 55, 60, 65, 70, 75, and 80 µM) were used. The final concentration of DMSO in the culture medium did not exceed 0.1% (v/v). Vehicle control cells received an equivalent volume of DMSO without kaempferol. Fresh working solutions were prepared immediately before each treatment.

### 2.3 Radioactive materials

Technetium-99 m radionuclide ^99m^Tc was obtained from a^99^Mo/^99m^Tc generator at the Clinical Nuclear Medicine Department, Mubarak Al-Kabeer Hospital, Kuwait.

### 2.4 Preparation of [^99m^Tc]Tc-PSMA

PSMA I&T kit obtained from the Center of Molecular Research (Moscow, Russia) was reconstituted under aseptic conditions following the manufacturer’s instructions, including quality control (QC), and radiochemical purity (RCP). Then the labeling efficiency was calculated. Briefly, 1–2 mL of freshly eluted sodium pertechnetate solution (30 mCi or 1110 MBq) was added to a sterile reaction vial containing the PSMA ligand and a reducing agent, typically stannous chloride (SnCl_2_.2H_2_O). The reaction mixture was gently mixed and incubated at room temperature.

The RCP of the prepared [^99m^Tc]Tc-PSMA was assessed using instant thin-layer chromatography (ITLC). Silica gel strips (ITLC-SG) were used. Two solvent systems were employed: acetone to determine free pertechnetate ([^99m^Tc]TcO_4_^−^), and saline to identify hydrolyzed-reduced technetium ([^99m^Tc]TcO_2_). After development, strips were counted using a dose calibrator.

(Capintec, United States). Radiochemical purity was calculated as the percentage of bound [^99m^Tc]Tc-PSMA relative to total radioactivity.

### 2.5 *In vitro* methodology

#### 2.5.1 Cell lines and culture medium

Human breast carcinoma Michigan Cancer Foundation-7 (MCF-7) cell lines were procured from Cytion (Lausanne, Switzerland). Stock cells were then cultured in Dulbecco’s Modified Eagle Medium (DMEM) media containing 10% fetal bovine serum (FBS) and 1% penicillin/streptomycin at 37 °C in 5% CO_2_.

#### 2.5.2 Cell culture and treatment

MCF-7 breast cancer cells were seeded in 12-well plates at a density of 5 × 10^5^ cells/well and allowed to adhere overnight. To evaluate the cytotoxic potential of the compounds, cells were treated with varying concentrations of kaempferol for 48 h.

#### 2.5.3 Cell viability assay (MTT assay)

Cell viability was assessed using the methyl thiazolyl tetrazolium (MTT) assay. MCF-7 cells were seeded into 96-well plates and treated in triplicate with kaempferol at concentrations ranging from 0 to 80 µM (0, 5, 10, 15, 20, 25, 30, 35, 40, 45, 50, 55, 60, 65, 70, 75, and 80 µM) for 48 h. Following treatment, the culture medium was removed and replaced with MTT solution (0.5 mg/mL in phosphate-buffered saline [PBS]). Cells were incubated for 2 h at 37 °C to allow the formation of purple formazan crystals by metabolically active cells. The MTT solution was then carefully removed, and the resulting formazan crystals were dissolved in 200 µL of dimethyl sulfoxide (DMSO). Absorbance was measured at 560 nm using a microplate reader (BioTek Instruments, VT, United States). Blank wells containing culture medium and MTT reagent without cells were included for background correction. Cell viability was expressed as a percentage relative to untreated control cells (0 µM kaempferol), which were considered 100% viable.

For IC_50_ determination, concentration–response data obtained from individual replicate measurements were analyzed using nonlinear four-parameter logistic regression. The fitted concentration–response curve was used to estimate the half-maximal inhibitory concentration (IC_50_), and the corresponding 95% confidence interval (CI) was calculated from the nonlinear regression analysis.

#### 2.5.4 Flow cytometry analysis of apoptosis

Apoptosis was evaluated using the Guava Nexin® assay according to the manufacturer’s instructions. MCF-7 cells (1 × 10^6^ cells) were cultured and treated in triplicate with kaempferol at concentrations of 50, 100, and 150 µM for 48 h. Positive and negative control groups were included in each experiment. Following treatment, cells were suspended in medium containing 1% FBS. For staining, 100 µL of each cell suspension was transferred to a 1.5-mL microcentrifuge tube, and 100 µL of Guava Nexin Reagent was added. Samples were gently mixed and incubated for 20 min at room temperature in the dark. The percentages of viable, early apoptotic, late apoptotic, and necrotic cells were subsequently determined using the Guava flow cytometry system.

#### 2.5.5 *In vitro* wound healing assay

Cell migration was evaluated using an *in vitro* wound healing (scratch) assay. MCF-7 cells were seeded in 12-well plates at a density of 1 × 10^4^ cells/well and cultured until a confluent monolayer was formed. A linear wound was then created across the confluent cell monolayer using a sterile pipette tip. Detached cells and cellular debris were removed by gently washing with phosphate-buffered saline (PBS).

Fresh culture medium containing kaempferol at the selected concentrations (60 μM and 80 µM) was subsequently added, while untreated cells served as the control group. The selected concentrations were based on the MTT cell viability results and nonlinear dose-response analysis. Cells were incubated for 24 and 48 h, and migration into the wound area was monitored using an optical microscope at ×200 magnification. Images were captured immediately after scratch induction (0 h) and after 24 and 48 h of incubation.

The extent of wound closure was quantified using Image-Pro Plus software (Media Cybernetics, MD, United States). Cell migration was expressed as the percentage of wound closure relative to the initial wound area. All experiments were performed in triplicate and independently repeated three times.

### 2.6 *In vivo* methodology

#### 2.6.1 Ethics statement

All animal experiments were conducted in accordance with institutional guidelines and approved by the appropriate ethics committee. The experiments were ethically approved by the ethical standards, guidelines, and instructions set by the Guide for the Care and Use of Laboratory Animal Resources Centre of Kuwait University, College of Medicine, Animal Resources Centre, and the approval by the scientific local ethics committee (Approval number MF-24–17). Rats were maintained under controlled conditions (12-h dark/light cycle, 24 °C, 45% humidity) with fed with normal pellet *ad libitum* food and water.

#### 2.6.2 DMBA-induced mammary tumor model and experimental design

Thirty 8-week-old female rats were randomly allocated into three experimental groups (n = 10 per group). Mammary tumor induction was initiated by administration of a single dose of 7,12-dimethylbenz [a]anthracene (DMBA; 80 mg/kg body weight) dissolved in sunflower oil. DMBA was administered in a final volume of 0.4 mL by oral gavage during Week 3. Therefore, no injection site was applicable.

Group I served as the cancer control group and received DMBA only. In Group II (pre-treatment group), kaempferol was administered orally at a dose of 100 mg/kg/day from Week 1 to Week 12, with DMBA administered during Week 3. In Group III (post-treatment group), animals received DMBA during Week 3 followed by kaempferol treatment (100 mg/kg/day, oral gavage) from Week 7 to Week 12.

Following completion of the treatment period, animals continued to be monitored for tumor development until Week 16, when imaging was performed. The experimental timeline has been clarified in Figure 1 and Table 1.

**FIGURE 1:**
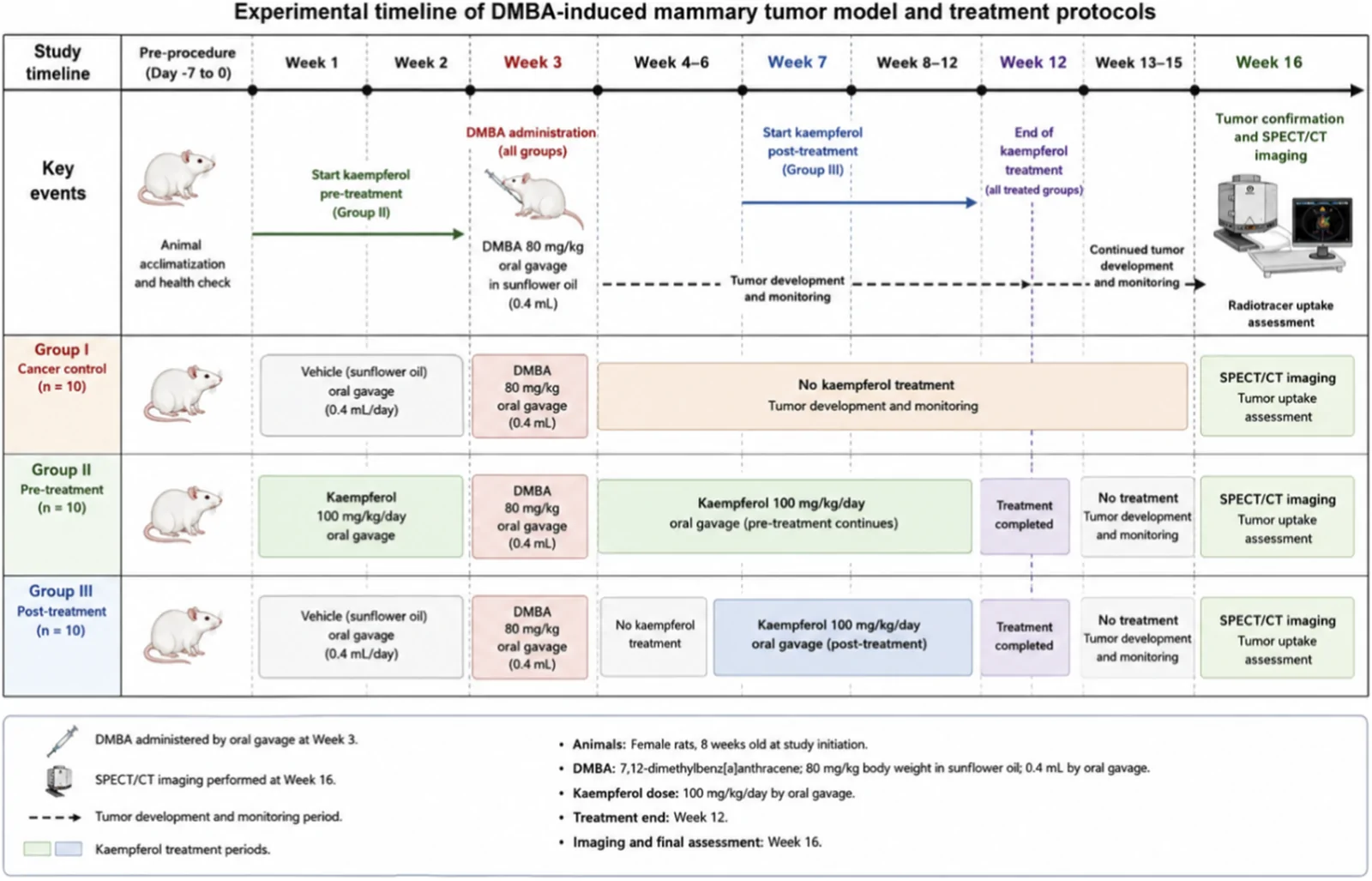
Experimental timeline for DMBA-induced mammary tumor development and SPECT/CT imaging. Rats were anesthetized before the experimental procedures, followed by DMBA administration according to the assigned experimental protocol. Animals were monitored for mammary tumor development throughout the study period. Kaempferol treatment was administered according to the pre-treatment and post-treatment schedules and concluded at Week 12. Tumor development was subsequently monitored until Week 16, when animals underwent SPECT/CT imaging for assessment of tumor-associated radiotracer uptake.

**TABLE 1:** Animal experimental protocol.

Group	Number of animals	Treatment protocol
Group I: Cancer control	10	A single oral dose of DMBA (80 mg/kg body weight) dissolved in sunflower oil was administered by oral gavage (0.4 mL) during week 3
Group II: Pre-treatment	10	Kaempferol (100 mg/kg/day) was administered by oral gavage (0.5 mL) from week 1 to week 12. A single oral dose of DMBA (80 mg/kg body weight in sunflower oil; 0.4 mL) was administered during week 3
Group II: Pre-treatment	10	Kaempferol (100 mg/kg/day) was administered by oral gavage (0.5 mL) from week 1 to week 12. A single oral dose of DMBA (80 mg/kg body weight in sunflower oil; 0.4 mL) was administered during week 3

The kaempferol dose (100 mg/kg/day, oral gavage) was selected based on previously published *in vivo* studies (Kaur et al., 2024; Imran et al., 2019; Qattan et al., 2022; Chen and Chen, 2013) demonstrating significant anticancer activity and acceptable safety profiles in rodent models. Doses within the range of 50–150 mg/kg have been shown to inhibit tumor progression, modulate apoptosis-related signaling pathways, and suppress angiogenesis without evidence of significant toxicity. Therefore, a dose of 100 mg/kg/day was considered appropriate for evaluating the therapeutic effects of kaempferol in the present breast cancer model.

#### 2.6.2 *In vivo* SPECT/CT imaging and image analysis

Rats from each experimental group were anesthetized using ketamine:xylazine (1:1 v/v) anesthetic drug cocktail. An amount of [^99m^Tc]Tc-PSMA 0.3 mL-0.5 mCi (18.5 MBq) was administered into the sedated animals intravenously via tail vein injection. Whole-body scans integrating the SPECT/CT functionalities were acquired immediately after injection, with an acquisition time of 20 min.

A quantitative measure and three-dimensional distribution of the injected [^99m^Tc]Tc-PSMA dose was done using NanoScan Trimodality small animal imaging system. This system contains whole body SPECT/CT imaging. Acquired images were processed by defining the circumference of tumor areas (T), and background such as soft tissues and whole-body regions (B). Quantitative analysis of tumor uptake was performed using tumor-to-background ratio (TBR) derived from region-of-interest (ROI) measurements.

The activities of the defined ROI’s are tabulated and the TBR is accounted in each case. The values calculated in the ROIs served as indicators of radiotracer retention. The key metric used is the ratio reflects how much tracer accumulates in the tumor region relative to the background whole body.

Quantification:

$$TBR=\frac{\text{Tumor}\;\text{uptake}}{\text{Background}\;\text{uptake}}$$

Quantitative image analysis was performed using ROI methodology. Tumor regions were identified based on areas of maximal signal intensity and manually delineated using consistent ROI sizes across all subjects. Background ROIs were drawn in adjacent non-tumor tissue to account for nonspecific signal.

#### 2.6.4 Western blot analysis of E-cadherin and VEGF expression

MCF-7 cells were treated with kaempferol at concentrations of 10, 20, 40, and 80 μM, while untreated cells served as the control group. Following treatment, cells were lysed using radioimmunoprecipitation assay (RIPA) buffer supplemented with protease and phosphatase inhibitors. Total protein concentrations were determined using a bicinchoninic acid (BCA) protein assay kit. Equal amounts of protein (20 µg per lane) were separated by sodium dodecyl sulfate-polyacrylamide gel electrophoresis (SDS-PAGE) and subsequently transferred onto polyvinylidene difluoride (PVDF) membranes.

Following blocking, the membranes were washed with phosphate-buffered saline (PBS) and incubated for 2 h at room temperature with the respective primary antibodies. E-cadherin expression was assessed using a mouse monoclonal anti-E-cadherin antibody (ab231303, 1:500 dilution; Abcam), whereas VEGF expression was assessed using a rabbit polyclonal anti-VEGF antibody (ab46154, 1:500 dilution; Abcam). β-Actin was detected using a rabbit monoclonal antibody (ab8227, 1:500 dilution; Abcam) and served as the internal loading control.

After incubation with the primary antibodies, the membranes were washed with PBS and incubated for 1 h at room temperature with the corresponding species-specific horseradish peroxidase (HRP)-conjugated secondary antibodies. E-cadherin was detected using a goat anti-mouse IgG (H + L) HRP-conjugated secondary antibody (1,706,516, 1:5,000 dilution; Bio-Rad Laboratories). VEGF and β-actin were detected using a goat anti-rabbit IgG H&L (HRP)-conjugated secondary antibody (ab205718, 1:5,000 dilution; Abcam).

Following secondary antibody incubation, the membranes were washed with PBS and developed using a chemiluminescent detection solution. Protein bands were visualized using the LuminoGraph II imaging system (Atto Corp., Japan). Band intensities were quantified using ImageJ software, and the expression levels of E-cadherin and VEGF were normalized to the corresponding β-actin signal.

### 2.7 Statistical analysis

Data are presented as mean ± standard deviation (SD). Statistical comparisons between groups were performed using one-way analysis of variance (ANOVA) followed by Tukey’s *post hoc* test. Normality was assessed using the Shapiro–Wilk test. Effect sizes were calculated to evaluate the magnitude of observed differences, with eta squared (η^2^) reported for ANOVA analyses and Cohen’s d reported for pairwise comparisons. Cohen’s d values were interpreted according to standard thresholds: small (0.2), medium (0.5), and large (0.8). A p value <0.05 was considered statistically significant.

## 3 Results

### 3.1 *In vitro* findings

#### 3.1.1 Preparation of [^99m^Tc]Tc-PSMA

After the preparation of [^99m^Tc]Tc-PSMA, the RCP of the prepared [^99m^Tc]Tc-PSMA was calculated to be 98%. The RCP of ≥95% was considered acceptable for further biological applications. The final preparation was tested for pH (acceptable range: 4.5–7.5) using pH indicator strips. Sterility and endotoxin testing were performed according to pharmacopeial guidelines when intended for *in vivo* use.

#### 3.1.2 Cell viability assay (MTT assay)

Kaempferol significantly reduced MCF-7 cell viability in a concentration-dependent manner (p < 0.01). Cell viability progressively declined from 100% in untreated control cells to 81.50% ± 0.10% at 20 μM, 60.23% ± 0.21% at 35 μM, and 50.00% ± 0.21% at 40 μM, with further reductions observed at higher concentrations, reaching 21.03% ± 0.15% at 80 µM. Nonlinear four-parameter logistic regression analysis of the triplicate concentration-response data estimated an IC_50_ of 40 µM (95% CI: 35.93–43.96 µM).

#### 3.1.3 Flow cytometry analysis of apoptosis

Figure 2 represents the apoptotic analysis using annexin V assay, the MCF-7 cells showed minimal apoptotic levels at low doses of <150 µM of kaempferol. But MCF-7 cells increased early apoptosis (11.4%) and reduced viable cells at higher concentrations reaching 150 µM.

**FIGURE 2:**
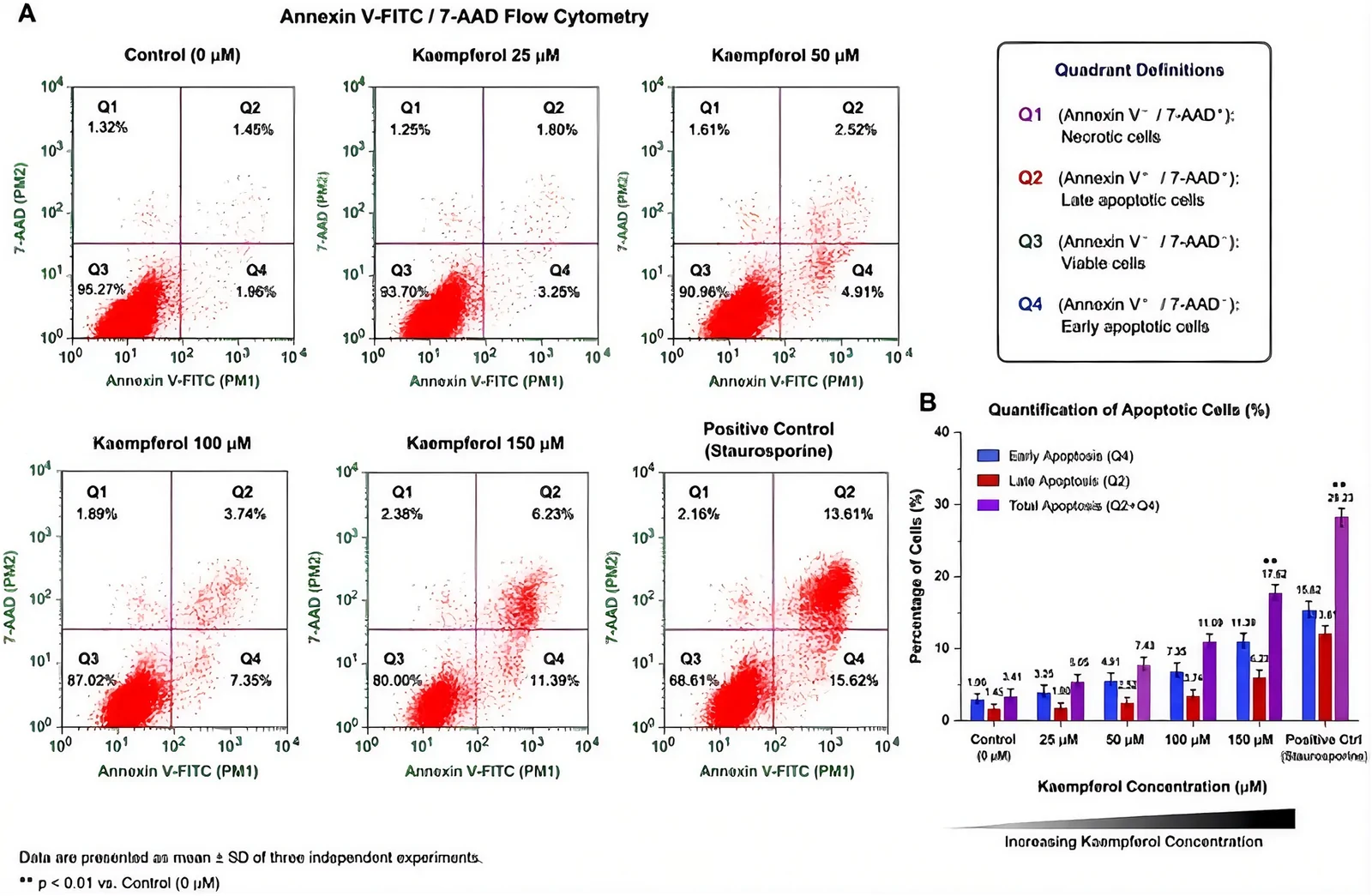
Flow cytometric analysis of apoptosis in MCF-7 cells after kaempferol treatment using Annexin V-FITC/7-AAD staining. Left: gating strategy. Right: apoptosis distribution. Kaempferol induced minimal apoptosis at concentrations <150 µM (∼2%), whereas 150 µM significantly increased early apoptotic cells (∼11.4%), demonstrating a dose-dependent effect. Cell populations: Q1, necrotic; Q2, late apoptotic; Q3, viable; Q4, early apoptotic. **(A)** Representative flow cytometry plots. **(B)** Quantification of apoptotic cells. **p < 0.01 vs. control.

In apoptosis assay control cells show a baseline level of apoptosis and necrosis. The kaempferol concentration of 25–100 µM treatments appear to maintain cell viability and reduce late-stage cell death. While 150 µM kaempferol treatment increases early apoptosis and reduces viability.

#### 3.1.4 *In vitro* wound healing assay

The wound healing assay demonstrated time- and concentration-dependent effects of kaempferol treatment on cell migration at 24 and 48 h (Figure 3).

**FIGURE 3:**
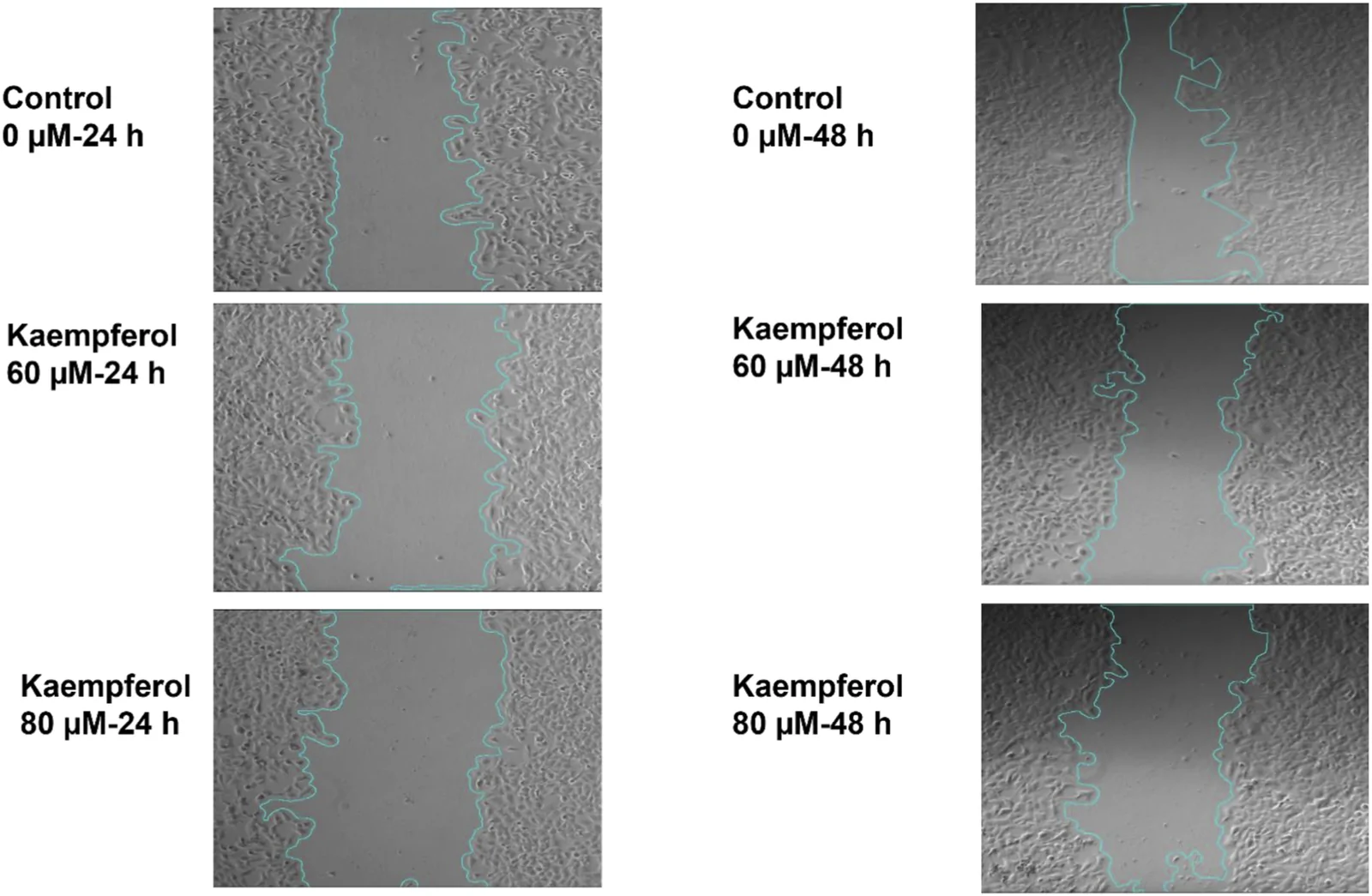
Wound healing assay showing the effects of kaempferol on MCF-7 cell migration at 24 and 48 h. Kaempferol inhibited migration in a time- and concentration-dependent manner, with 80 µM producing the greatest reduction in wound closure (p < 0.001) compared with controls.

In the control group, cells exhibited progressive migration into the wound area over time. At 24 h, partial closure of the scratch was observed, while at 48 h, a substantial reduction in wound width indicated active and continuous cell migration.

In contrast, treatment with kaempferol at 60 μM and 80 µM resulted in a noticeable inhibition of wound closure. At 24 h, both concentrations showed reduced migration compared to control, with wider remaining gaps between wound edges. This inhibitory effect became more pronounced at 48 h, where the wound area remained significantly larger than in control samples.

Among the treated groups, kaempferol 80 µM exhibited the strongest inhibitory effect (p < 0.001 versus control (p < 0.05), with minimal progression of wound closure between 24 and 48 h.

The kaempferol 60 µM group showed moderate inhibition, with some migration occurring but still markedly less than control.

The outlined wound boundaries (cyan tracings) confirm these observations, showing reduced edge advancement in treated groups compared to controls.

#### 3.1.5 *In vivo* SPECT/CT imaging and image analysis

Figure 4 represents three-dimensional whole-body images illustrating tumor localization and signal intensity. Rats were randomly divided into three groups (A) cancer control (DMBA only) (B) pre-treatment (kaempferol administered prior to DMBA exposure), and (C) post-treatment (kaempferol administered after tumor induction). Visual ROI is determined. Then quantitative ROI analysis of tracer uptake across experimental groups was performed using Tumor-to-background ratio (TBR). Data are presented as mean ± SD with individual data points (n = 10 per group, Table 2).

**FIGURE 4:**
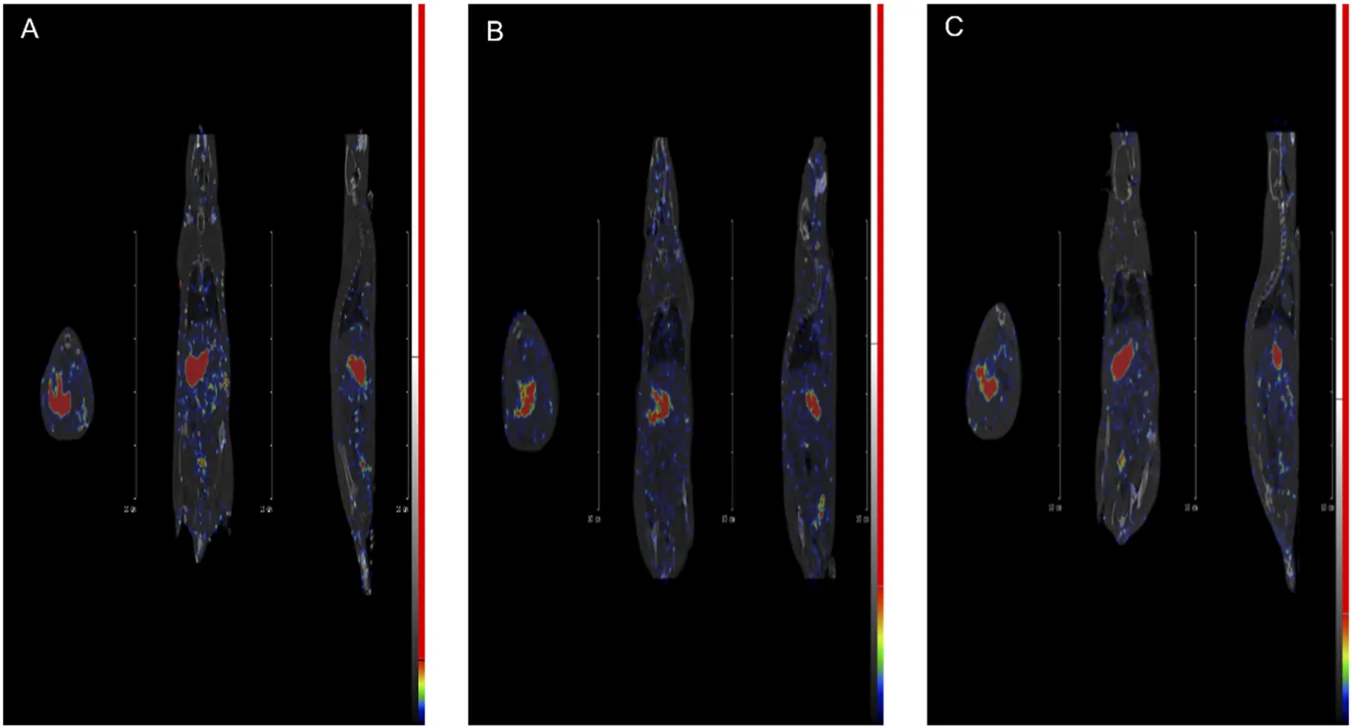
SPECT/CT *in vivo* imaging of DMBA-induced tumors in Sprague–Dawley rats following kaempferol intervention. Groups: **(A)** cancer control (DMBA only), **(B)** pre-treatment (kaempferol before DMBA), and **(C)** post-treatment (kaempferol after tumor induction). Representative 3D whole-body images (left: isolated tumor; middle: coronal; right: sagittal) show tumor localization and signal intensity. n = 10/group.

**TABLE 2:** Tumor-to-Background Ratio (TBR) quantification. The results are expressed by mean ± SD, *p < 0.05 versus control.

TBR/Rat	Group a	Group B	Group C
1	71.1	65.5	70.2
2	68.4	63.2	71.1
3	73.2	67.8	70.8
4	71.3	66.1	71.1
5	71.5	64.9	71.6
6	69.9	62.7	69.7
7	71.2	66.1	72.4
8	68.7	63.2	71.3
9	74.2	63.8	70.2
10	72.3	63.2	73.2
Mean ± SD	71.18 ± 1.83	*64.65 ± 1.69	71.16 ± 1.06

Group B demonstrated significantly reduced tumor uptake compared to Groups A and C (**p < 0.01). No significant difference (ns) was detected between Groups A and C. Statistical analysis was performed using one-way ANOVA followed by *post hoc* multiple comparisons.

Quantitative analysis of tumor uptake was performed using TBR derived from ROI measurements. Group A demonstrated a mean TBR of 71.18 ± 1.83, while Group B showed a significantly reduced uptake (64.65 ± 1.69, *p < 0.05) compared to A and C. C Group exhibited similar TBR (71.16 ± 1.06) as A with no significant difference was observed between Groups A and C.

Tumor-to-background ratio (TBR) analysis demonstrated a significant difference among the three experimental groups (one-way ANOVA, F (2.27) = 58.09, p = 1.66 × 10^−10^, η^2^ = 0.81). The mean TBR was 71.18 ± 1.83 in Group A, 64.65 ± 1.69 in Group B, and 71.16 ± 1.06 in Group C. Tukey’s *post hoc* analysis revealed that TBR was significantly lower in Group B compared with Group A (mean difference = 6.53, adjusted p < 0.001**) and Group C (mean difference = −6.51, adjusted p < 0.001**), whereas no significant difference was observed between Groups A and C (mean difference = 0.02, adjusted p = 0.99). The corresponding effect sizes indicated very large differences between Groups A and B (Cohen’s d = 3.71) and Groups C and B (Cohen’s d = 4.62), while the difference between Groups A and C was negligible (Cohen’s d = 0.01). These findings indicate that the reduction in tumor-associated tracer uptake was specifically observed in the pre-treatment Group B, whereas the post-treatment Group C remained comparable to the cancer control Group A (Figure 5).

**FIGURE 5:**
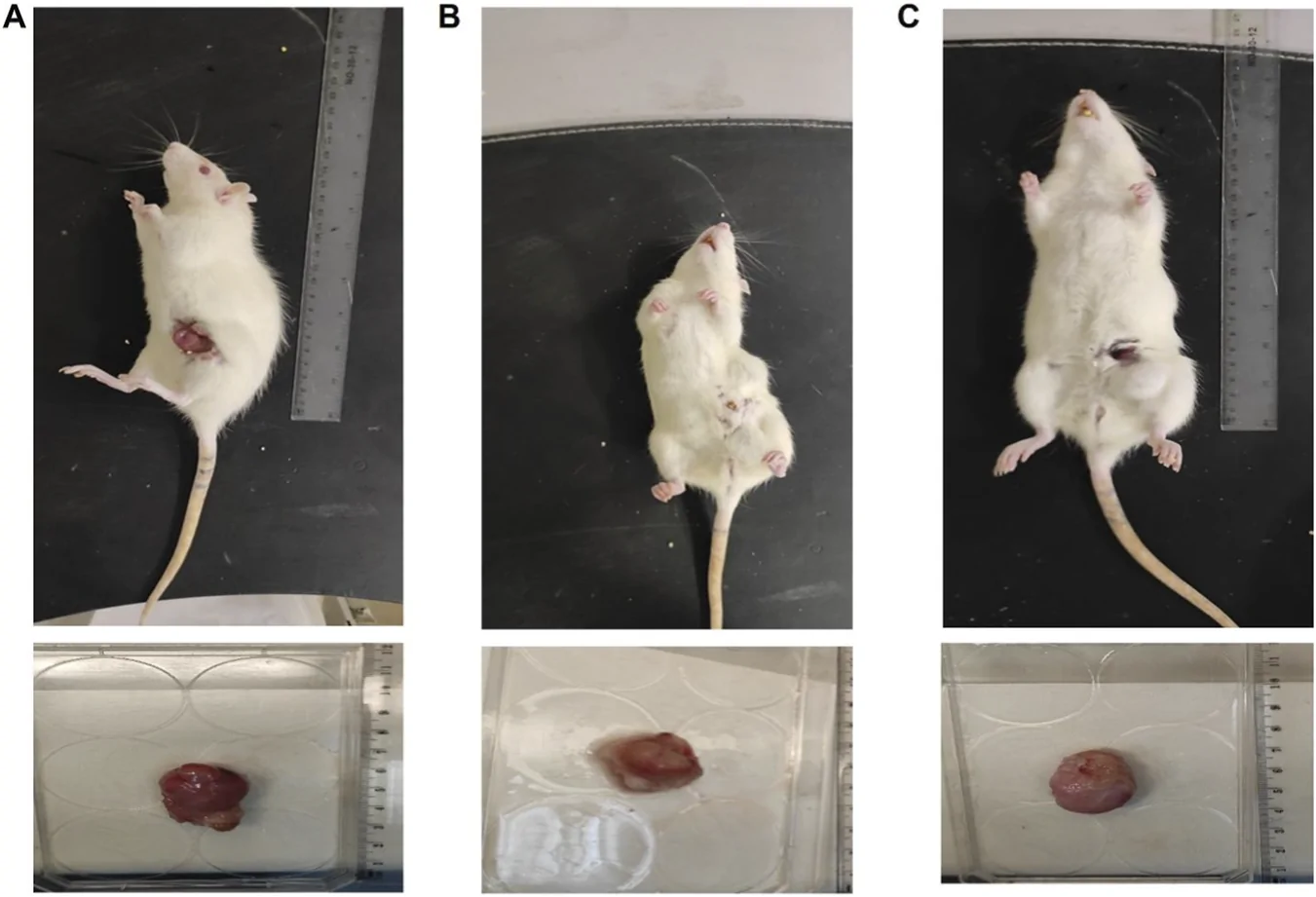
Representative images of tumor development and excision in the SD rat model. Upper panels **(A–C)**: dorsal views showing tumor growth on the flank region. Lower panels: tumor burden comparison, with Panels A and C showing larger tumors and Panel B showing a smaller tumor mass. Scale indicated by ruler.

#### 3.1.6 Effect of kaempferol on EMT-related protein expression

Western blot analysis demonstrated that kaempferol markedly modulated the expression of epithelial-to-mesenchymal transition (EMT)-related proteins in MCF-7 cells (Figure 6). As shown in Figure 6, E-cadherin expression increased progressively following kaempferol treatment, whereas VEGF expression decreased in a concentration-dependent manner compared with untreated control cells. In contrast, the expression of β-actin remained constant across all experimental groups. Densitometric analysis of the immunoblots revealed a significant upregulation of E-cadherin and a concomitant downregulation of VEGF in kaempferol-treated cells compared with the control group. The changes in protein expression were statistically highly significant (P < 0.001) and were most pronounced at the highest kaempferol concentration.

**FIGURE 6:**
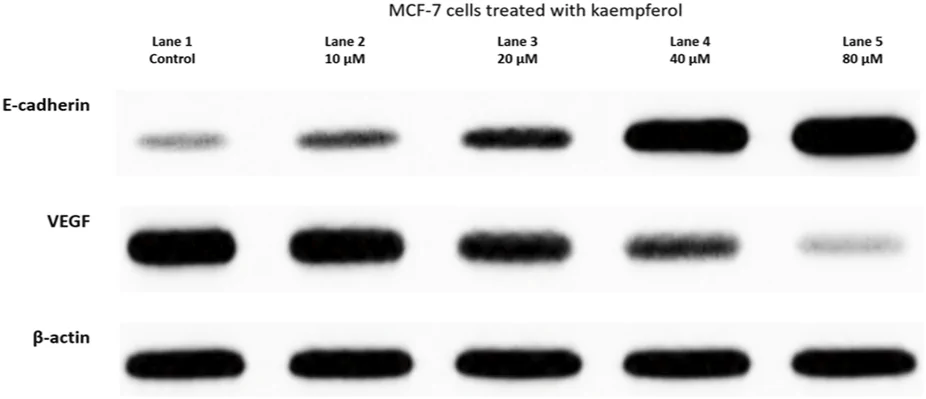
Effects of kaempferol on E-cadherin and VEGF protein expression in MCF-7 cells. Representative Western blots and densitometric analyses demonstrate concentration-dependent upregulation of E-cadherin and downregulation of VEGF following treatment with kaempferol (0–80 µM). Protein expression was normalized to β-actin. Data are presented as mean ± SD from independent experiments. Statistical analysis was performed using one-way ANOVA followed by Tukey’s multiple-comparison test. *p < 0.05, **p < 0.01, and ***p < 0.001 versus untreated control.

## 4 Discussion

The present study investigated the potential anticancer effects of kaempferol using complementary *in vitro* and *in vivo* approaches, including MCF-7 cell viability and wound-healing assays, Western blot analysis of selected molecular markers, and [^99m^Tc]Tc-PSMA SPECT/CT imaging in a DMBA-induced breast cancer model. Overall, the findings demonstrate concentration-dependent effects of kaempferol on MCF-7 cell viability, modulation of E-cadherin and VEGF expression, and differences in tumor-associated tracer uptake among the experimental groups. However, the results should be interpreted according to the specific endpoints directly measured in the present study.

The MTT assay demonstrated a concentration-dependent reduction in MCF-7 cell viability following kaempferol treatment. Reanalysis of the concentration-response data using nonlinear four-parameter logistic regression yielded an estimated IC_50_ of 40 µM (95% CI: 27.7–47.5 µM). These findings confirm that kaempferol exerted a measurable concentration-dependent cytotoxic effect on MCF-7 cells under the experimental conditions used. Previous studies have similarly reported antiproliferative effects of kaempferol in different cancer models (Sandhu et al., 2025; Sarwar et al., 2018; Li M et al., 2025; Zhu and Xue, 2019; Al-Saeedi and Rajendran, 2024; Santana et al., 2012; Shahid et al., 2026). Nevertheless, the present experiment was designed to evaluate cell viability and does not independently establish the specific molecular pathways responsible for the observed cytotoxicity.

The wound-healing assay demonstrated reduced wound closure in kaempferol-treated MCF-7 cells, suggesting an inhibitory effect on the ability of cells to repopulate the wounded area. However, this finding should be interpreted with caution. The concentrations used in the wound-healing experiment included concentrations that may affect cell viability, particularly in relation to the experimentally determined IC_50_. Therefore, reduced wound closure cannot be attributed exclusively to inhibition of cell migration and may partially reflect reduced cell proliferation and/or cytotoxic effects. Consequently, the present results should not be considered definitive evidence of a specific anti-migratory or anti-metastatic effect of kaempferol. Future studies should repeat the assay using concentrations associated with minimal cytotoxicity and include an appropriate proliferation control, such as mitomycin C. Complementary migration and invasion assays, including Transwell-based approaches, would also be valuable to clarify the direct effects of kaempferol on cancer cell motility.

Western blot analysis demonstrated changes in the expression of E-cadherin and VEGF following kaempferol treatment. These changes are consistent with previous reports demonstrating that kaempferol contributes to restoration of E-cadherin expression and suppression of VEGF-mediated signaling pathways in cancer models (Qattan et al., 2022; Jo et al., 2015; Wei and Zhang, 2024; de Morais et al., 2024; Felice et al., 2022; Haroon and Kang, 2025; Li et al., 2026; Avila-Carrasco et al., 2019; Carrano et al., 2024; Deng et al., 2026). Increased E-cadherin expression may indicate modulation of cell-cell adhesion, whereas decreased VEGF expression suggests a potential effect on angiogenesis-related signaling. However, these findings should be interpreted within the limits of the measured endpoints. Because additional epithelial-mesenchymal transition (EMT) markers, angiogenic mediators, and functional angiogenesis assays were not included, the observed changes do not establish complete EMT inhibition or direct inhibition of angiogenesis. Similarly, molecular pathways including PI3K/AKT/mTOR, NF-κB, N-cadherin, MMPs, and other signaling mediators were not experimentally investigated in the present study. Their potential involvement should therefore be considered literature-based hypotheses rather than mechanisms demonstrated by the present findings.

The following diagram (Figure 7) illustrates that kaempferol exerts its anticancer effects primarily through the inhibition of key oncogenic signaling pathways, notably the PI3K/AKT/mTOR axis and the NF-κB pathway. In this mechanism, kaempferol suppresses PI3K activation, which subsequently reduces downstream phosphorylation of AKT and mTOR, leading to decreased cellular proliferation, protein synthesis, and survival signaling. Concurrently, kaempferol inhibits NF-κB activation by preventing the degradation of its inhibitor IκBα, thereby reducing the transcription of pro-inflammatory and pro-survival genes. These molecular effects collectively contribute to the modulation of EMT, a critical process in cancer metastasis. Specifically, kaempferol downregulates mesenchymal markers such as N-cadherin while upregulating epithelial markers like E-cadherin, resulting in reduced cell migration, invasion, and metastatic potential. Overall, these coordinated actions lead to suppression of tumor progression and enhancement of apoptotic processes.

**FIGURE 7:**
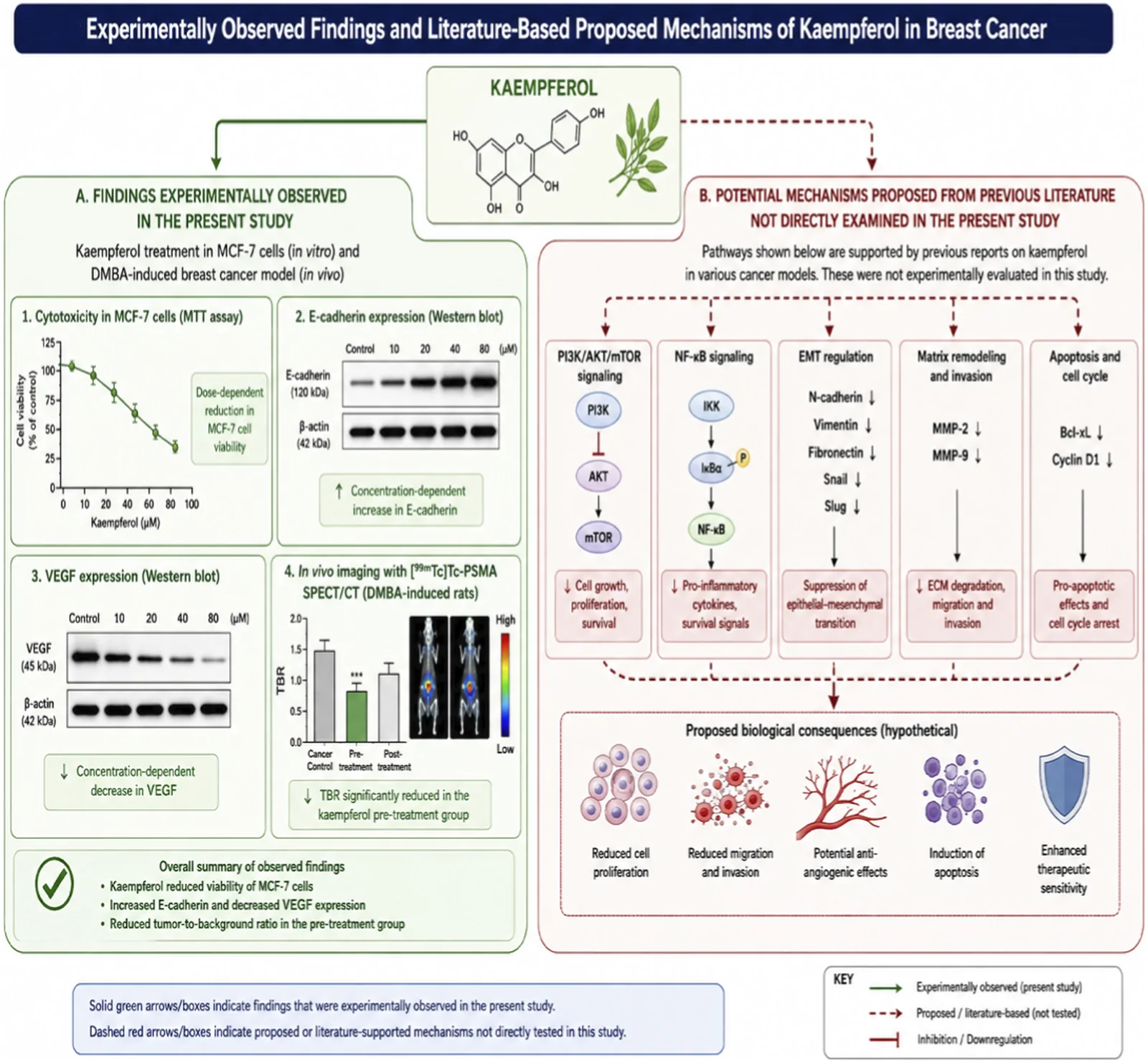
Experimentally observed findings and literature-based proposed mechanisms of kaempferol in breast cancer. The experimentally observed findings of the present study are highlighted separately and include concentration-dependent cytotoxicity in MCF-7 cells, increased E-cadherin expression, decreased VEGF expression, and reduced tumor-to-background ratio (TBR) in the kaempferol pre-treatment group. The potential involvement of PI3K/AKT/mTOR signaling, NF-κB signaling, epithelial-mesenchymal transition (EMT), matrix metalloproteinases, apoptosis-related proteins, and other molecular pathways is based on findings reported in previous studies and was not directly examined in the present investigation. Dashed arrows indicate proposed or literature-supported mechanisms rather than experimentally demonstrated effects. Therefore, the schematic should be interpreted as a hypothesis-generating mechanistic model.

The *in vivo* imaging results provide additional information regarding the biological effects associated with kaempferol administration in the DMBA-induced breast cancer model. Reanalysis of the tumor-to-background ratio (TBR) data demonstrated a statistically significant overall difference among the experimental groups. The pre-treatment group showed significantly lower TBR values compared with both the cancer control and post-treatment groups, whereas no significant difference was observed between the cancer control and post-treatment groups. These findings indicate that administration of kaempferol before DMBA exposure was associated with reduced tumor-associated [^99m^Tc]Tc-PSMA uptake. In contrast, kaempferol administration after tumor induction did not result in a measurable reduction in tracer uptake compared with the cancer control group.

This pattern should be interpreted cautiously and is more consistent with a possible chemopreventive or tumor-modifying effect than evidence of therapeutic activity against established tumors. Therefore, the present results do not support a conclusion that kaempferol effectively treats established breast cancer under the experimental conditions used. In addition, reduced radiotracer uptake alone cannot be interpreted as direct evidence of tumor regression, reduced tumor burden, or therapeutic efficacy. Further studies incorporating longitudinal tumor-volume measurements, tumor multiplicity, final tumor weight, histopathological confirmation, and molecular characterization are required to establish the biological significance of the observed changes in tracer uptake.

The use of [^99m^Tc]Tc-PSMA SPECT/CT imaging represents a potentially useful non-invasive approach for monitoring changes in tumor-associated tracer uptake during experimental interventions. However, the interpretation of PSMA-targeted tracer uptake in a non-prostatic tumor model requires particular caution. Alterations in tracer uptake may reflect changes in multiple biological factors and cannot independently establish changes in tumor size or therapeutic response.

The present findings are in agreement with previous studies highlighting the potential of [^99m^Tc]Tc-PSMA imaging to enhance the diagnosis and management of non-prostatic cancers (Dall' Armellina et al., 2025).

Future studies combining serial molecular imaging with conventional tumor measurements and histopathological analysis would provide a more comprehensive understanding of the relationship between tracer uptake and tumor biology.

Several limitations of the present study should also be acknowledged. The animal experiment lacked healthy, vehicle-treated, kaempferol-only, and standard-treatment control groups, limiting the ability to distinguish preventive, therapeutic, and nonspecific effects. Furthermore, comprehensive longitudinal data on tumor incidence, multiplicity, tumor volume, final tumor weight, histopathological confirmation, body-weight changes, and systemic toxicity were not available for all experimental groups. These limitations restrict the interpretation of kaempferol’s therapeutic efficacy in the DMBA-induced breast cancer model. Future studies should incorporate these parameters together with blinded outcome assessment, predefined exclusion criteria, and clearly defined humane endpoints.

Another challenge in the potential therapeutic development of kaempferol is its limited aqueous solubility and oral bioavailability. Formulation-based approaches, including nanoformulations, lipid-based carriers, and cyclodextrin-based delivery systems, may offer strategies to improve the pharmaceutical performance of poorly soluble phytochemicals. (Pyrak et al., 2025) discussed cyclodextrin nanosponges as potential bioenhancers for poorly soluble plant-derived compounds. Although this approach has not been specifically validated for kaempferol in the cited work, it provides a relevant formulation framework that could be explored in future studies to improve kaempferol solubility and bioavailability.

Overall, the present findings demonstrate that kaempferol exerts concentration-dependent effects on MCF-7 cell viability and modulates the expression of E-cadherin and VEGF. The reduction in wound closure suggests a potential effect on cell motility; however, because cytotoxicity and proliferation were not independently controlled in this assay, a migration-specific effect cannot be conclusively established. In the DMBA-induced animal model, reduced [^99m^Tc]Tc-PSMA uptake was observed in the kaempferol pre-treatment group, whereas the post-treatment group was not significantly different from the cancer control group. Collectively, these findings suggest that kaempferol may have biological and potentially chemopreventive effects under the experimental conditions examined. However, further well-controlled studies incorporating comprehensive tumor characterization, functional migration and invasion assays, mechanistic investigations, pharmacokinetic evaluation, optimized formulations, and appropriate therapeutic control groups are required before definitive conclusions regarding its anticancer efficacy can be drawn.

## 5 Conclusion

In conclusion, kaempferol was associated with concentration-dependent cytotoxic effects in MCF-7 cells and modulation of cancer-related molecular markers *in vitro*. In the DMBA-induced mammary tumor model, reduced tumor-associated radiotracer uptake was observed predominantly in animals receiving kaempferol before tumor development, whereas post-treatment did not significantly differ from the cancer control group. These findings suggest a potential chemopreventive or tumor-modulatory role for kaempferol rather than demonstrated therapeutic efficacy against established tumors. Further studies incorporating comprehensive tumor burden measurements, histopathological confirmation, appropriate control groups, and toxicity assessments are required to validate these preliminary observations.

## Data Availability

The original contributions presented in the study are included in the article/supplementary material, further inquiries can be directed to the corresponding author.
